# Ten year experience with antiretroviral treatment in Cambodia: Trends in patient characteristics and treatment outcomes

**DOI:** 10.1371/journal.pone.0185348

**Published:** 2017-11-14

**Authors:** Phirum Lay, Sokkab An, Sunpiseth Soeung, Pich Sovannary Srey, Sopheak Thai, Lutgarde Lynen, Johan van Griensven

**Affiliations:** 1 Sihanouk Hospital Center of HOPE, Phnom Penh, Cambodia; 2 Institute of Tropical Medicine, Antwerp, Belgium; National and Kapodistrian University of Athens, GREECE

## Abstract

**Background:**

Although HIV disease stage at ART initiation critically determines ART outcomes, few reports have longitudinally monitored this within Asia. Using prospectively collected data from a large ART program at Sihanouk Hospital Center of Hope in Cambodia, we report on the change in patient characteristics and outcomes over a ten-year period.

**Methods:**

We conducted a retrospective analysis including all adults (≥ 18 years old) starting ART from March 2003-March 2013 in a non-governmental hospital in Phnom Penh, Cambodia. The cumulative incidence of death, lost to follow-up (LTFU), attrition (death or LTFU) and first line treatment failure were calculated using Kaplan-Meier methods. Independent risk factors for these outcomes were determined using Cox regression modeling.

**Results:**

Over the ten-year period, 3581 patients initiated ART with a median follow-up time of 4.8 years (IQR 2.8–7.2). The median age was 35 years (IQR 30–41), 54% were female. The median CD4 count at ART initiation increased from 22 cells/μL (IQR 4–129) in 2003 to 218 (IQR 57–302) in 2013. Over the 10 year period, a total of 282 (7.9%) individuals died and 433 (12.1%) were defined LTFU. Program attrition (died or LTFU) was 11.1% (95% CI: 10.1%- 12.4%) at one year, 16.3% (95% CI: 15.1%-17.6%) at three years, 19.8% (95% CI: 18.5%-21.2%) at five years and 23.3% (95% CI: 21.6–25.1) at ten years. Male sex and low baseline body mass index (BMI) were associated with increased attrition.

Factors independently associated with mortality included a low baseline CD4 count, older age, male sex, low baseline BMI and hepatitis B co-infection. Individuals aged above 40 years old had an increased risk of mortality but were less likely to LTFU.

There were a total of 137 individuals with first line ART failure starting second line treatment. The probability of first line failure was estimated at 2.8% (95% CI: 2.3%-3.4%) at 3 years, 4.6% (95% CI: 3.9%-5.5%) at 5 years and 7.8% (95% CI 4.8%-12.5%) at ten years of ART. The probability was particularly high in the first few program years. A lower risk was observed among individuals starting ART during the 2006–2008 period. Factors independently associated with an increased risk of treatment failure included ART-experience, NVP-based ART and a baseline CD4 count below 200 cells/μL.

**Conclusions:**

Overall program outcomes were fair, and generally compare well to other reports from the region. Despite gradually earlier initiation of ART over the ten year period, ART is still initiated at too low CD4 count levels, warranting increased efforts for early HIV diagnosis and enrolment/retention into HIV care. Tailored strategies for poor prognostic groups (older age, male, low BMI) should be designed and evaluated.

## Introduction

There are currently around 36.7 million individuals infected with HIV globally, of which 17 million were on antiretroviral treatment (ART) representing only 46% of those in need [1]. In 2003, the World Health Organization (WHO) issued its first ART guidelines, recommending a public health approach for scaling-up of ART in low and middle income countries. Over the subsequent ten years, crucial changes have occurred, including the availability of cheap generic antiretrovirals (ARVs) and increased financial resources to combat HIV/AIDS. Most low income countries have achieved successful ART scaling-up, with universal coverage in several [1]. Moreover, at regular intervals, the WHO guidelines have been updated following an evidence-based approach, with research findings feeding into guidelines and policy. One of the major changes in these guidelines relates to the CD4 count threshold to initiate ART. As low CD4 counts at ART initiation has a strong association with subsequent mortality [2, 3], the guidelines have evolved to gradually increasing levels, from 200 cells/μL to 350 cells/μL and 500 cells/μL in the 2003, 2010 and 2013 guidelines respectively [4–7]. The 2016 guidelines recommend universal treatment for all HIV-positive individuals regardless of CD4 count at HIV diagnosis [8].

There are however few ART program reports that have adopted a long term perspective, describing the implementation of the subsequent changes, the evolution in patient characteristics and outcomesand the long-term results in a routine clinical care setting. While a few such studies have been published long-term program results in sub-Saharan Africa [9–11], data are particularly scarce from the Asiaand Pacific setting, which hosts 5.1 million HIV infected individuals [1].

Cambodia is a low-income country in South-East Asia, with an estimated population of 15 million. Initially, Cambodia was among the most HIV affected Asian countries. After reaching 2.4% in 1998, the HIV prevalence decreased markedly to 0.6% in 2016 and the incidence decreased over tenfold over the same period [12]. Reportedly, the coverage of ART for adults was around 78.9% in 2014 [12].

Using standardized routinely collected data from a large ART program in Cambodia, we report on the 1) changes in patient characteristics over time; 2) CD4 evolution over time after ART initiation; 3) time to death, lost to follow-up and attrition (death or LTFU) while on ART; 4) time to first-line ART failure, in relation to different program periods. Factors associated with the different outcomes are reported as well.

## Methods

### Study setting

The Sihanouk-Hospital-Center-of-Hope (SHCH) in Phnom Penh, Cambodia is a non-governmental hospital located in the capital Phnom Penh. Since March 2003, the hospital provides comprehensive HIV care free of charge, as part of the national ART program. The main mission of the hospital is to provide quality care for the poorest of the poor. The hospital caters for patients originating from across the country, with around half coming from outside the capital. The hospital also acts as a research and training center, aiming to conduct relevant research that can enhance the evidence-base for clinical practice and can foster rational use of available resources. From the onset of the HIV care program, standardized data collection tools were developed in collaboration with the Institute of Tropical Medicine-Antwerp. Clinical guidelines–building on the WHO and national guidelines—were established for the SHCH.

### Study design and study population

We conducted a retrospective cohort study enrolling all adult (≥ 18 years old) HIV-infected patients initiating ART at SHCH between March 2003 and March 2013. Those that had started on ART in another center and subsequently presenting at SHCH while taking ART (transfer-ins) were excluded.

### Antiretroviral treatment initiation and monitoring

**Fig 1:**
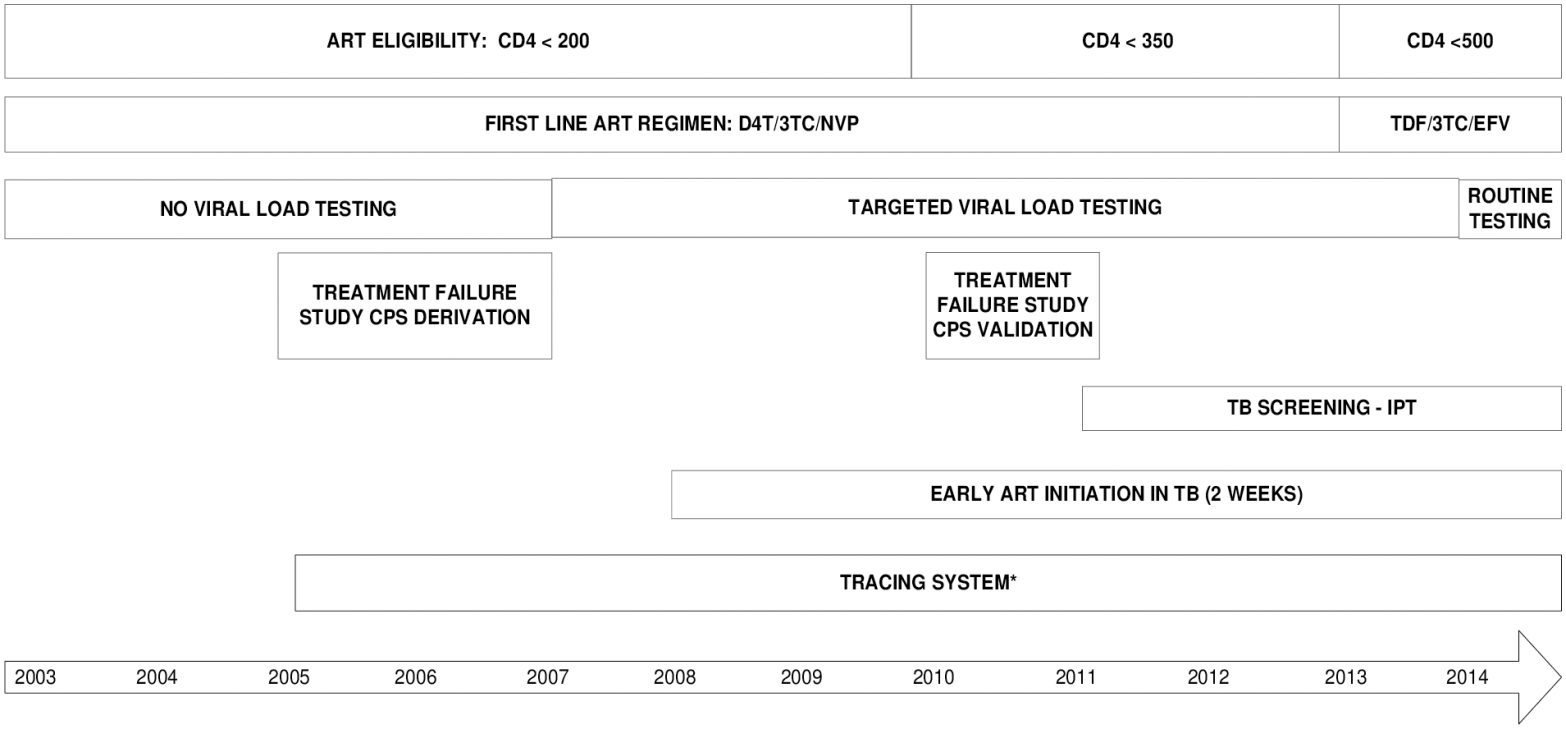
Overview of key changes occurring in the HIV program (2003–2014). WHO: World Health Organization, ART: Antiretroviral therapy, CPS: clinical prediction score, IPT: INH preventive tuberculosis, TDF: tenofovir, 3TC: lamivudine, EFV: efavirenz, NVP: nevirapine, D4T: stavudine, TB: tuberculosis. * Clerks or counselors call patients who were not present on the day of follow up.

As per WHO guideline, the eligibility criteria have evolved over time, as summarized in Fig 1. For the entire study period, standard first line treatment consisted of a generic fixed dose combination containing stavudine, lamivudine and nevirapine. In case of contraindications to stavudine or nevirapine, zidovudine or efavirenz was prescribed.

During the pre-ART preparatory work-up, all patients received extensive counselling before ART and concurrent opportunistic infections were ruled out or treated when detected. Patients were seen at two and four weeks after starting ART, followed by monthly visits. After the first six months of ART, visits were scheduled less frequently (every 2–3 months) for clinically stable patients. All medical care was provided by physicians, supported by a team of nurses and adherence counsellors. At every clinical encounter, a number of key issues were systematically addressed, including the assessment of treatment response and ART-related toxicity. Adherence assessment relied on pill counts (at every visit), and the visual analogue scale (every six months). Baseline laboratory testing included haematology, liver function tests, hepatitis B/C testing and CD4 cell count determination (FACSCount (Becton Dickinson). For hepatitis B infection, hepatitis B surface antigen was determined. Hepatitis C diagnosis relied on antibody detection. Both tests were done using a chemiluminescence immunoassay (CIA) on a Cobas e 411 analyzer (Roche Diagnostics, Mannheim Germany) from 2009 on, and an AxSYManalyzer (Abbott laboratories, Illinois, US) before that. After ART initiation, a full blood count and CD4 cell count was done every six months. Liver function tests were done at month 1, 2, 3, 6 after ART initiation, followed by six-monthly measurements. Details of the ARV program have been published previously [13–17].

Monitoring strategies to detect first-line treatment failure have also evolved over time (Fig 1). In the first years of the program, no viral load testing could be done. Between November 2005 and May 2007, a cross-sectional study was conducted, to develop a clinical prediction score for targeted viral load testing, and viral load samples were sent abroad [18]. Subsequently, viral load testing could be done in Cambodia. From 2007 on, this clinical prediction score has been used in the program, and viral load testing could be done within the national program for individuals clinically or immunologically failing first-line ART. A study to validate the clinical prediction score was conducted between May 2010 and June 2011 [19]. From June 2014 on, the national program recommends routine viral load testing. From 2003 to 2012, a cut-off value of 5000 copies/ml was used to switch from first line to second line antiretroviral therapy. From 2013 on, a cut-off of 1000 copies/ml was used.

Cotrimoxazole prophylactic treatment was given for all WHO stage II/III/IV patients and all those with a CD4 count < 200 cells⁄μL. From October 2008 on, all patients with WHO stage IV disease or a CD4 count < 100 cells⁄μL were started on fluconazole primary prophylaxis and were screened for cryptococcalantigenemia [20]. Previous ART experience defines as patients had taking ART before presenting to our hospital, but had stopped taking it when presenting at our hospital. Patients missing their appointment and not presenting at the hospital for a period of three months were contacted by phone every day for four times then every month. If living within Phnom Penh or the surrounding areas, home visits were organized. Those we failed to contact after six months were declared lost to follow-up.

For patients dying outside the hospital, death ascertainment relied on the LTFU tracing system, with the date of death provided by the family members, as the phone would usually still be used (by a family member).

#### Outcomes and operational definitions

There were three main outcomes: 1) CD4 evolution over time after ART initiation; 2) time to death, lost to follow-up and attrition (death or LTFU) while on ART; 3) time to first-line ART failure defined as any individuals started on second line ART (i.e. protease inhibitor-based) with “first line treatment failure” mentioned as indication in the database.

Three periods were identified since the launch of the ART program: 2003–2006 (the early phase of the program and scaling-up), 2007–2010 (maturing of the program) and 2011–2013 (a period of budgetary and operational constraints for HIV care at the national level). By 2011, several international non-governmental organizations providing ARThad handed over their activities. Coinciding with some budgetary constraints at the national level, this was also the time that some centers occasionally re-started restricting enrolment of new patients. SHCH remained providing HIV care free of charge during that period, without restricting enrolment.

#### Data collection and statistical analysis

Clinical and laboratory data werecollected on a daily basis, using paper-based standardized data collection tools and stored electronically in a database. All physicians were systematically trained in the use of the case definitions and patient management according to the hospital guidelines. ART-toxicity grading followed WHO recommendations. Quality control of the stored data was done at regular intervals. The data are available as supporting information (S1 Data).

Baseline patient characteristics were described and compared using χ2 or Fisher’s exact tests for categorical variables and the Kruskal-Wallis test for continuous variables. In descriptive analysis, the median CD4 count and IQRs were calculated at baseline and at six monthly intervals after ART initiation. In addition, we calculated the median time spent with CD4 counts below 350 cells/μL, per program period using Kaplan-Meier methods (time to reaching a CD4 count > 350 cells/μL after ART initiation, excluding those with a CD4 count > 350 cells/μL at baseline). Time to reaching a CD4 count > 350 cells/μL across the different periods was compared using the log-rank test. We also used Kaplan-Meier methods for the time to event analyses with death, LTFU, attrition or first line ART treatment failure as outcome. Person-time at risk was calculated, starting from the date of ART initiation up to either the date of the event of interest, date of death, date of last visit for those LTFU or transferred-out, and March 2013 for the remainder. Independent risk factors for ART failure were determined using multivariate Cox regression, including the following *a priori* defined factors: ART-experience, age, sex, baseline CD4 count, WHO clinical stage, baseline body weight, first line ART regimen (D4T vs non D4T; NVP vs non-NVP), program period. All these predefined factors were retained in the multivariate model, no back-ward selection process was done. The proportional hazard assumption was assessed graphically and tested formally using Schoenfeld residuals. In all analyses, biologically plausible interactions were evaluated. Collinearity was evaluated by calculating the variance inflation factors. Data were analyzed using STATA version 11 (STATACorp LP, College Station, United States of America). The level of significance was set at P<0.05.

#### Ethical issues

Since the launch of the HIV care program, clinical data have been routinely collected for purposes of program monitoring and evaluation, and research activities. Patients were requested to give written informed consent to store and use the data. No linkage of these data with other sources was done. The data collection and informed consent procedure were approved by the institutional review board of the SHCH and Institute of Tropical Medicine, Antwerp, Belgium. No patient identifiers were included in the dataset used for this analysis.

## Results

### Baseline characteristics

Between 2003 and 2013, a total of 5642 adult patients enrolled in HIV care at the hospital, of which 3581 started ART at the hospital and were eligible for inclusion in the study. The median age was 35 years (IQR 30–41), 54% were female. The median CD4 count at ART start was 97 cells/μL (IQR 26–222), 40.8% (1436/3520) had a body mass index < 18.5 kg/m^2^ at ART start. A total of 162 (4.5%) patients had used ARVs prior to ART initiation at SHCH but had discontinued these by the time they presented at the hospital (a history of ARV use). The most common initially prescribed regimen comprised stavudine, lamivudine and nevirapine, given to 2348 (66%) individuals. The median follow-up time on ART was 4.8 years (IQR 2.4–7.2), with a total follow-up of 17178 patient years. A total of 1240 individuals started ART between 2003 and 2006, 1762 were initiated between 2007 and 2010 and 579 started ART between 2011 and March 2013 (Table 1).

**Table 1:** Baseline characteristics of adults starting antiretroviral treatment(2003–2013; N = 3581).

	Total	2003–2006	2007–2010	2011–2013	P
Patients starting ART, n	3581	1240	1762	579	
Age (yrs), median (IQR)	35 (30–41)	34 (30–40)	35 (29–41)	36 (30–42)	<0.01
Female sex, n (%)	1936 (54.0)	614 (49.5)	987 (56)	335 (57.8)	<0.01
Previous ART exposure, n (%)	162 (4.5)	53 (4.3)	66 (3.7)	43 (7.4)	<0.01
Baseline CD4 cell count (cells/μL), Median (IQR)	97 (26.5–222.5)	53 (15–154)	133 (39–235)	176 (37–309)	<0.01
<50; n (%)	1297 (37.0)	598 (48.7)	525 (30.1)	174 (31.2)	
50–200; n (%)	1180 (33.4)	196 (15.9)	239 (13.7)	52 (9.3)	<0.01
201–350; n (%)	892 (25.3)	123 (10)	211 (12.1)	38 (6.8)	
>350; n (%)	159 (4.5)	188 (15.3)	606 (34.7)	257 (46)	
WHO stage, n (%)					<0.01
I and II	823 (23.0)	137 (11.5)	502 (28.4)	184 (31.8)	
III	1424 (40.0)	479 (38.6)	733 (41.6)	212 (36.6)	
IV	1334 (37.2)	624 (50.3)	527 (29.9)	183 (31.6)	
Advanced HIV disease at ART start, n (%)*	2138 (60.0)	840 (67.7)	781 (44.3)	385 (40.6)	
ART regimen, n (%)					<0.01
d4T/3TC/EFV	949 (26.5)	228 (18.4)	545 (30.9)	176 (30.4)	
d4T/ 3TC/NVP	2348 (65.5)	911 (73.5)	1111 (63.0)	326 (56.3)	
AZT/3TC/EFV	78 (2.2)	37 (3.0)	15 (0.8)	26 (4.5)	
AZT/3TC/NVP	165 (4.6)	63 (5.1)	78 (4.4)	24 (4.1)	
Other	41 (1.1)	1 (0.08)	13 (0.7)	27 (4.7)	

* Defined as a CD4 count < 100 cell/μL or a WHO clinical stage IV.

WHO: World Health Organization, ART: Antiretroviral therapy, TDF: tenofovir, 3TC: lamivudine, EFV: efavirenz, NVP: nevirapine, D4T: stavudine, AZT: zidovudine

#### Evolution in CD4 count at baseline and after ART initiation

**Fig 2:**
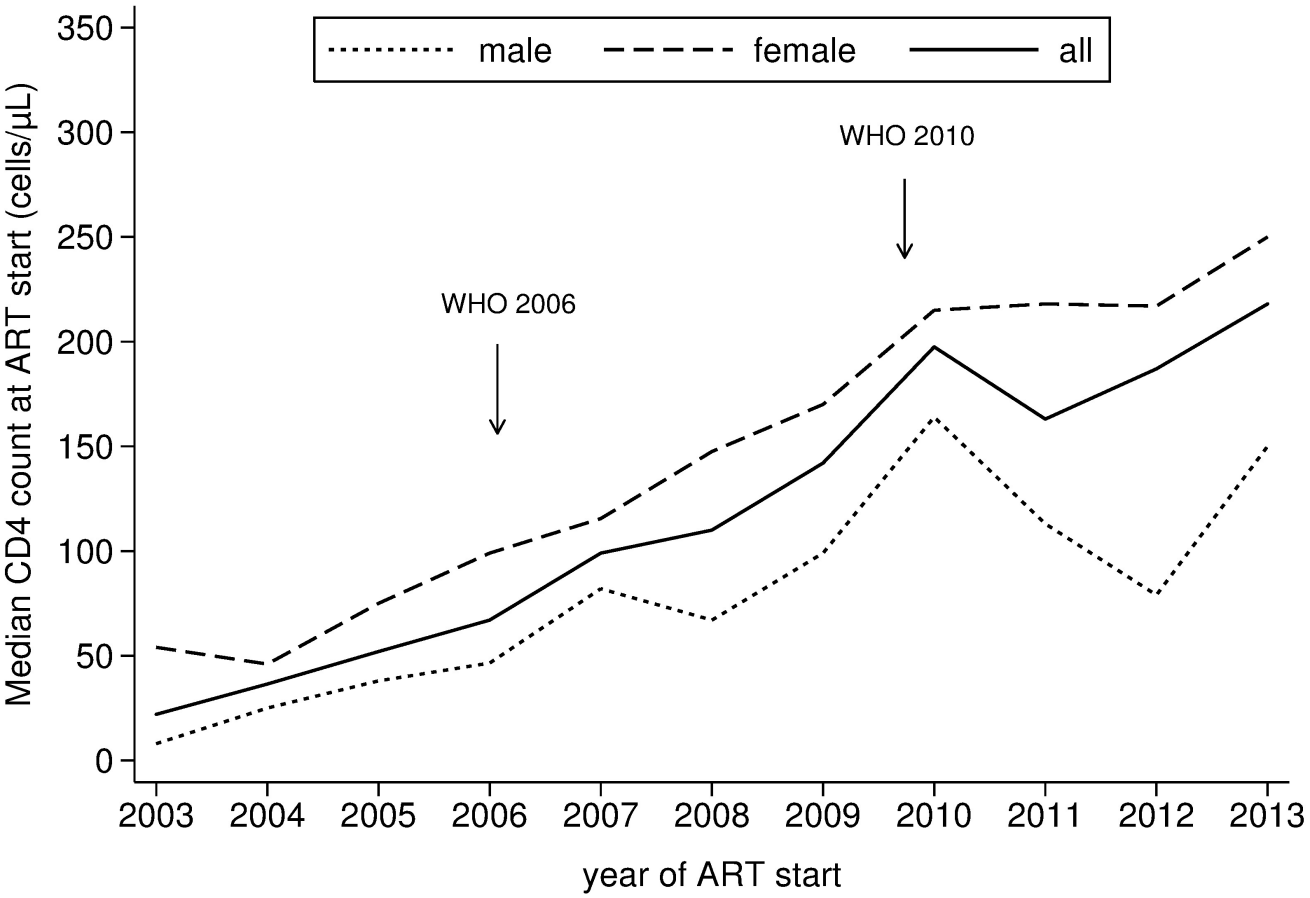
Evolution in baseline CD4 cell counts in adults initiating antiretroviral treatment. The arrows indicate the time revised WHO guidelines were implemented.

**Fig 3:**
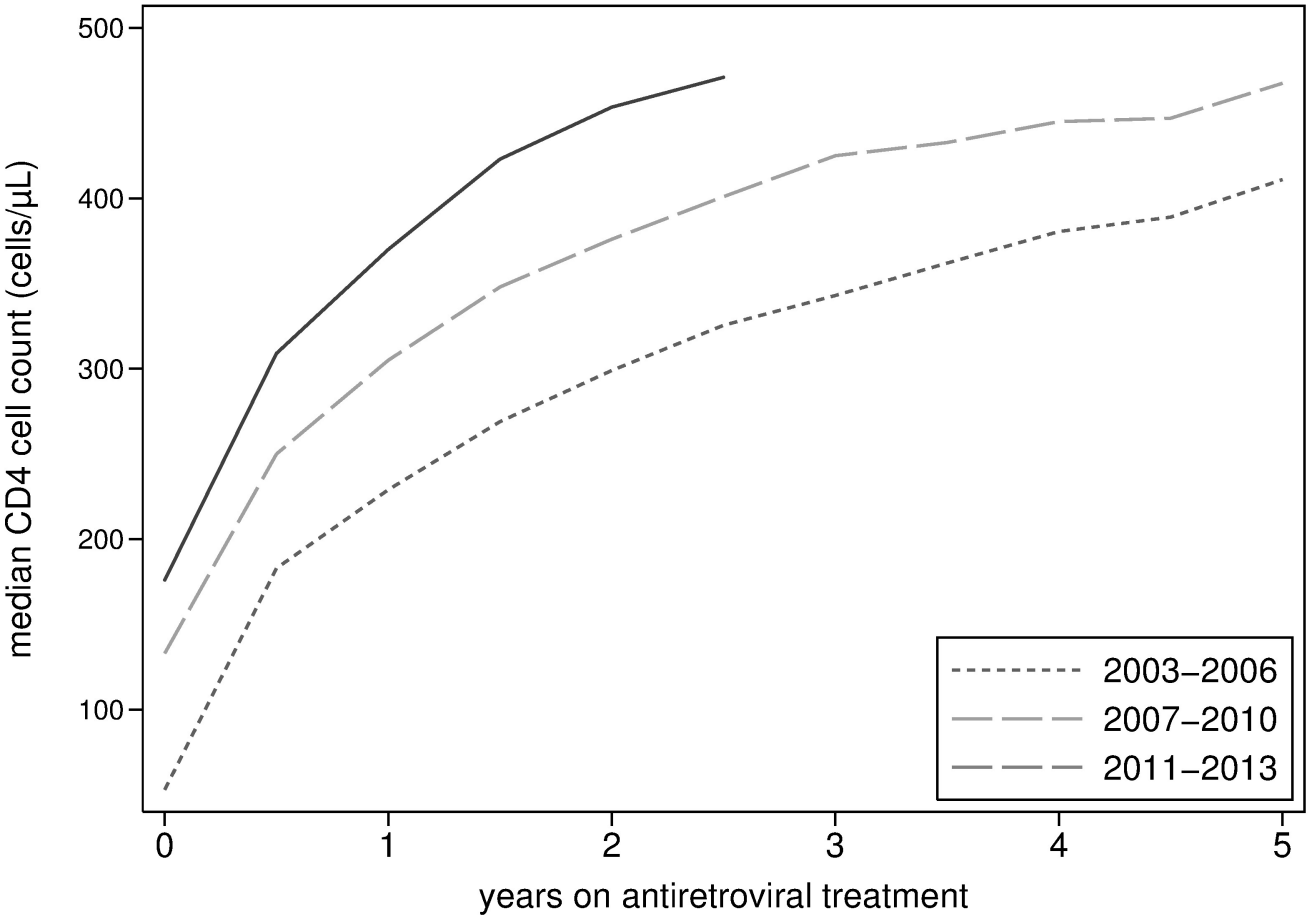
CD4 cell count evolution after initiation of antiretroviral treatment stratified by period (2003–2013).

The CD4 count at ART start increased gradually from 22 cells/μL (IQR 4–129) in 2003 to 218 cells/μL (IQR 57–302) in 2013 (Fig 2). Across the 10 year period, CD4 counts at ART initiation were consistently lower for men. A temporary decline in CD4 counts, particularly for men, was seen around 2011, a period when access to HIV care had decreased due to budgetary and operational constraints at the national level (see Methods). The evolution of the CD4 count while on ART is presented in Fig 3. The time spent on ART before reaching a CD4 count of 350 cells/μL decreased from 2.5 years (95% confidence interval (CI) 2.25–2.52) in 2003–2006, to 1.5 years (95% CI 1.48–1.51) in 2007–2010 and to 1.0 year (95% CI 0.99–1.01) in 2011–2013 (log-rank test: P value < 0.001).

#### Attrition, death and lost to follow-up

**Fig 4:**
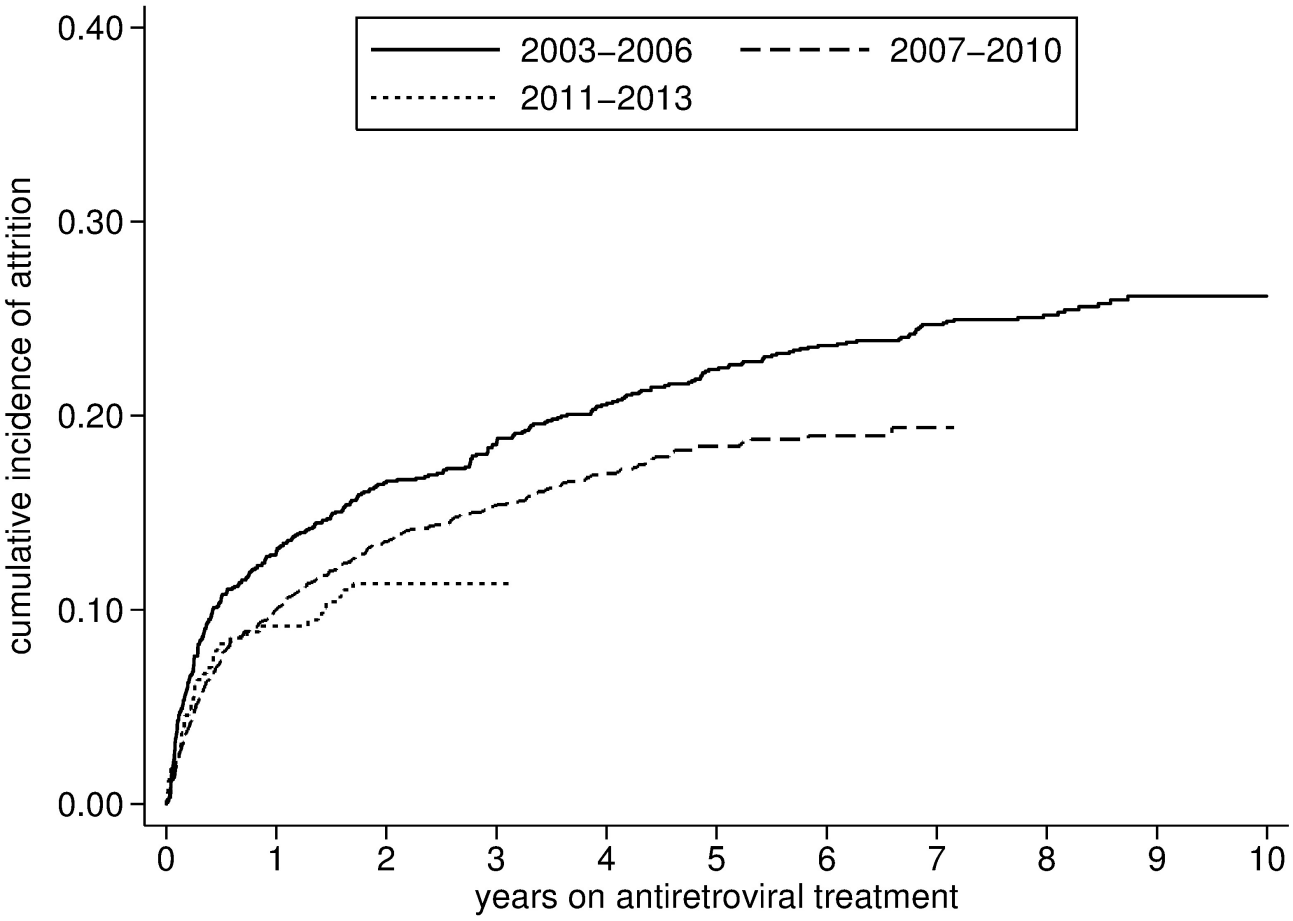
Attrition after initiation of antiretroviral treatment stratified by period (2003–2013).

**Fig 5:**
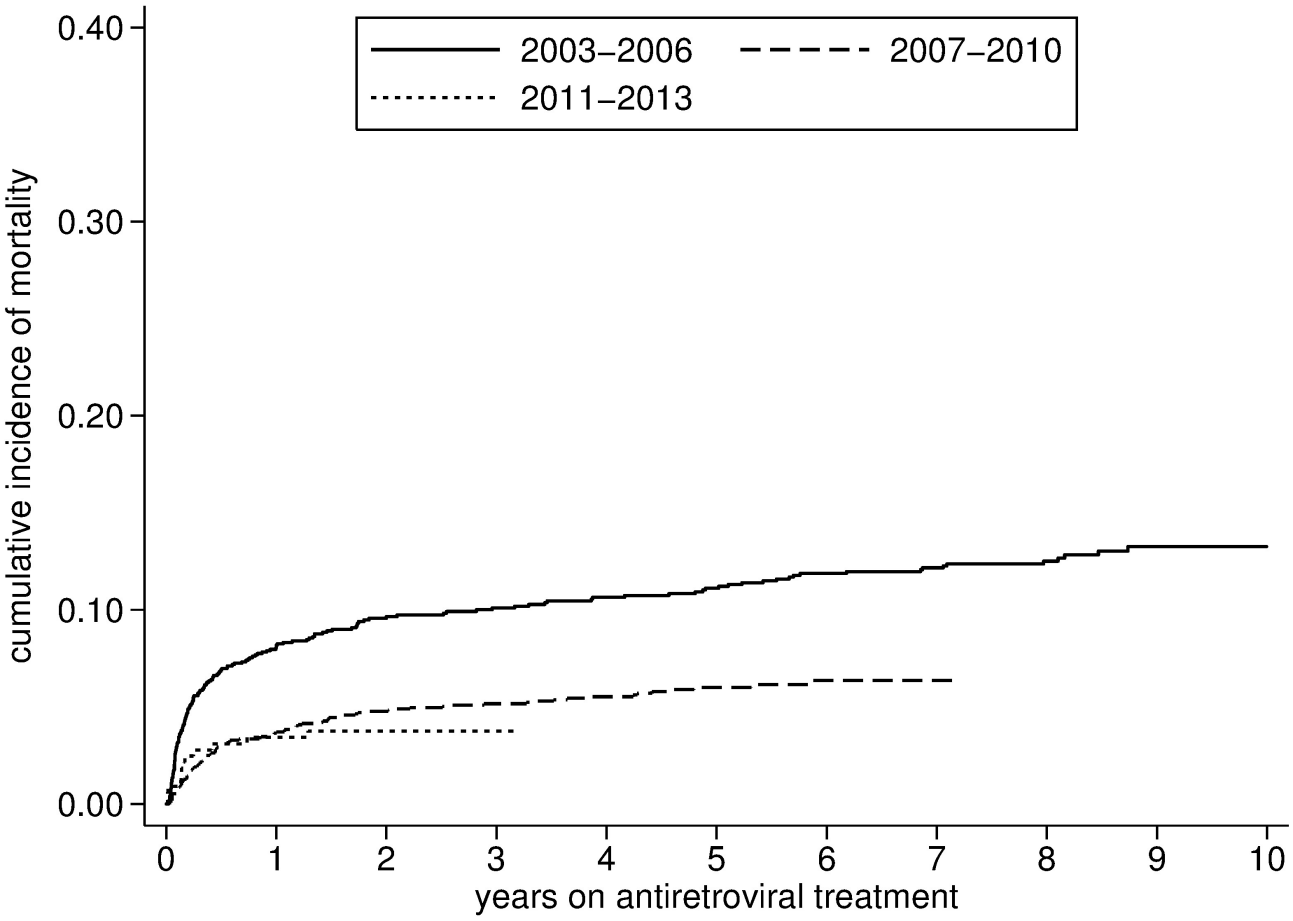
Mortality after initiation of antiretroviral treatment stratified by period (2003–2013).

**Fig 6:**
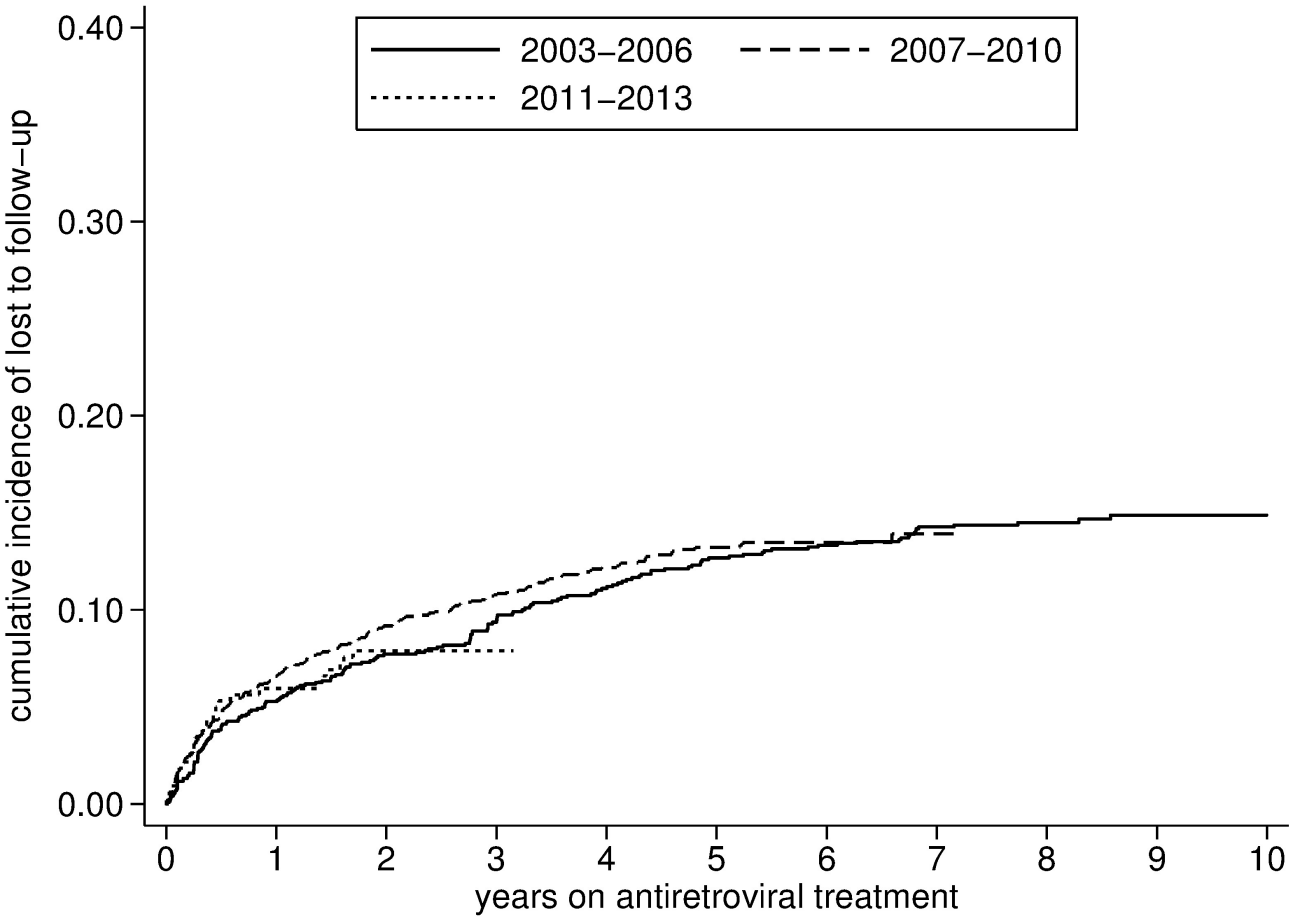
Lost to follow-up after initiation of antiretroviral treatment stratified by period (2003–2013).

Over the 10 year period, a total of 282 (7.9%) individuals died and 433 (12.1%) were defined LTFU. Attrition was estimated at 11.1% (95% CI: 10.1%-12.4%) at one year, 16.3% (95% CI: 15.1%-17.6%) at three years, 19.8%(95% CI: 18.5%-21.2%) at five years and 23.3% (95% CI: 21.6–25.1) at ten years. Mortality and attrition were particularly high during the early stages of the program (2003–2006) but subsequently stabilized (Figs 4 and 5). Conversely, the rate of lost to follow-up increased mildly after 2003–2006 (Table 2, Fig 6, and S1 Fig).

**Table 2:** Cumulative incidence of death, lost to follow-up and attrition at different time points after initiation of antiretroviral therapy (ART) overall and by gender and period of ART initiation (2003–2013; N = 3581).

Variable	1 year % (95% CI)	3 years % (95% CI)	5 years % (95% CI)	10 years % (95% CI)
All death	5.5 (5.0–6.3)	7.1 (6.3–8)	8.0 (7.1–9.0)	10.0 (9.0–11.4)
2003–2006	9.0 (7.4–10.6)	11.0 (9.1–12.7)	11.8 (10.1–14)	14.0 (12.0–16.1)
2007–2010	3.6 (3.0–4.6)	5.1 (4.2–6.3)	6.0 (5.0–7.3)	NA
2011–2013	3.9 (2.6–6.0)	4.5 (3.1–6.6)	NA	NA
Male	7.4 (6.3–9.0)	9.5 (8.1–11.0)	11.0 (9.3–12.5)	13.0 (11.0–15.0)
Female	4.0 (3.1–5.0)	5.0 (4.1–6.2)	5.6 (4.6–7.0)	7.6 (6.0–9.6)
All lost to follow-up	6.0 (5.2–7.0)	10.0 (9.0–11.0)	13.0 (11.6–14.1)	15.0 (13.4–16.3)
2003–2006	5.0 (4.0–6.2)	9.0 (7.3–10.6)	12.1 (10.3–14.2)	14.3 (12.4–16.6)
2007–2010	6.6 (5.5–8.0)	11.0 (9.4–12.3)	13.2 (11.6–15.0)	NA
2011–2013	6.6 (5.0–9.0)	9.0 (7.0–11.7)	NA	NA
Male	6.0 (5.0–7.0)	10.2 (9.0–12.0)	13.2 (11.5–15.1)	15.5 (13.3–18.0)
Female	6.1 (5.1–7.3)	9.7 (8.5–11.2)	12.5 (11.0–14.2)	14.2 (12.5–16.2)
All attrition	11.2 (10.2–12.2)	16.3 (15.2–17.6)	20 (18.5–21.2)	23.3 (21.6–25.1)
2003–2006	13.2 (11.5–15.3)	18.6 (16.6–21.0)	22.5 (20.3–25.0)	26.3 (24.0–29.0)
2007–2010	10.0 (8.6–11.5)	15.4 (14.0–17.1)	18.4 (16.6–20.4)	NA
2011–2013	10.3 (8.0–13.1)	13.0 (10.5–16.1)	NA	NA
Male	13.0 (11.3–14.5)	19.0 (17.0–21.0)	22.6 (20.5–25.0)	26.3 (24.0–29.0)
Female	10.0 (8.5–11.2)	14.3 (13.0–16.0)	17.4 (16.0–19.3)	21.0 (18.6–23.1)
All first line failure	0.0 (0.0–0.2)	3.0 (2.2–3.4)	4.6 (4.0–5.5)	8.0 (5.0–12.5)
2003–2006	0.0 (0.0–0.5)	4.1 (3.1–5.4)	6.0 (5.0–7.3)	9.0 (6.0–13.7)
2007–2010	0.0 (0.0–0.4)	1.4 (0.9–2.0)	3.1 (2.3–4.2)	NA
2011–2013	0.0 (NA)	NA	NA	NA
Male	0.1 (0–0.5)	3.4 (2.6–4.5)	5.1 (4.0–6.4)	6.0 (4.6–7.7)
Female	0.0 (NA)	2.2 (1.6–3.1)	4.2 (3.3–5.4)	9.4 (4.6–19.0)

Factors independently associated with mortality included a low baseline CD4 count, older age, male sex, a low baseline body mass index and hepatitis B co-infection. Individuals aged above 40 years old had an increased risk of mortality but were less likely LTFU (Table 3).

**Table 3:** Independent risk factors for death, lost to follow-up, attrition and first-line antiretroviral treatment failure (2003–2013; N = 3581).

	Death aHR (95% CI)	Lost to follow-up aHR (95% CI)	Attrition aHR (95% CI)	First line failure aHR (95% CI)
Age (years)				
≤ 40 years	Ref	Ref	Ref	Ref
> 40 years	1.72 (1.30–2.30)	0.72 (0.55–0.94)	1.03 (0.85–1.26)	0.70 (0.44–1.12)
Sex				
Female	Ref	Ref	Ref	Ref
Male	1.65 (1.25–2.18)	1.16 (0.93–1.44)	1.33 (1.12–1.57)	1.39 (0.97–2.00)
Previous ART experience				
No	Ref	Ref	Ref	Ref
Yes	0.90 (0.42–1.93)	0.71 (0.38–1.35)	0.79 (0.48–1.28)	3.80 (2.18–6.60)
Baseline body mass index				
≥18.5 kg/m2	Ref	Ref	Ref	Ref
<18.5 kg/m2	2.39 (1.78–3.20)	1.48 (1.19–1.85)	1.77 (1.48–2.10)	1.42 (0.98–2.05)
WHO clinical stage (baseline)				
I/II	Ref	Ref	Ref	Ref
III/IV	1.49 (0.92–2.41)	1.25 (0.92–1.70)	1.29 (1.0–1.67)	1.02 (0.62–1.68)
CD4 cell count category (cells/mL)				
≥200	Ref	Ref	Ref	Ref
<200	2.98 (1.85–4.81)	1.11 (0.85–1.44)	1.46 (1.16–1.82)	3.16 (1.78–5.60)
NRTI use				
D4T	Ref	Ref	Ref	Ref
Other	0.73 (0.44–1.22)	0.62 (0.43–0.90)	0.65 (0.48–0.88)	0.76 (0.40–1.54)
NNRTI use				
EFV	Ref	Ref	Ref	Ref
NVP	1.01 (0.75–1.37)	0.85 (0.67–1.08)	0.91 (0.76–1.10)	1.78 (1.12–2.83)
Period of ART start				
2003–2006	Ref	Ref	Ref	Ref
2007–2010	0.69 (0.51–0.93)	1.31 (1.03–1.67)	1.02 (0.85–1.23)	0.58 (0.39–0.87)
2011–2013	0.62 (0.38–1.03)	1.28 (0.87–1.88)	0.96 (0.71–1.30)	1.12 (0.53–2.33)
Hepatitis B co-infection				
No	Ref	Ref	Ref	Ref
Yes	1.54 (1.07–2.21)	1.04 (0.75–1.44)	1.22 (0.96–1.56)	0.85 (0.47–1.51)
Hepatitis C co-infection				
No	Ref	Ref	Ref	Ref
Yes	*_*	1.12 (0.70–1.80)	_	1.45 (0.70–3.02)

WHO: World Health Organization, aHR: adjusted hazard ratio, CI: confidence interval, Ref: reference, ART: Antiretroviral therapy, NNRTI: non-nucleoside analogue reverse transcriptase inhibitor, NRTI: nucleoside analogue reverse transcriptase inhibitor, TDF: tenofovir, 3TC: lamivudine, EFV: efavirenz, NVP: nevirapine, D4T: stavudine, AZT: zidovudine.

Male sex and a low baseline body mass index were positively associated with mortality, LTFU and attrition. Hepatitis B was associated with higher mortality. A baseline CD4 count below 200 cells/μL increased the risk of mortality and attrition. Compared to the first program years, later periods were independently–*i*.*e*. adjusted for the other variables–associated with lower mortality and higher LTFU(Tab 3). There were no significant associations with NVP use, but individuals started on a stavudine-containing regimen were less likely to be LTFU.

#### First line treatment failure

**Fig 7:**
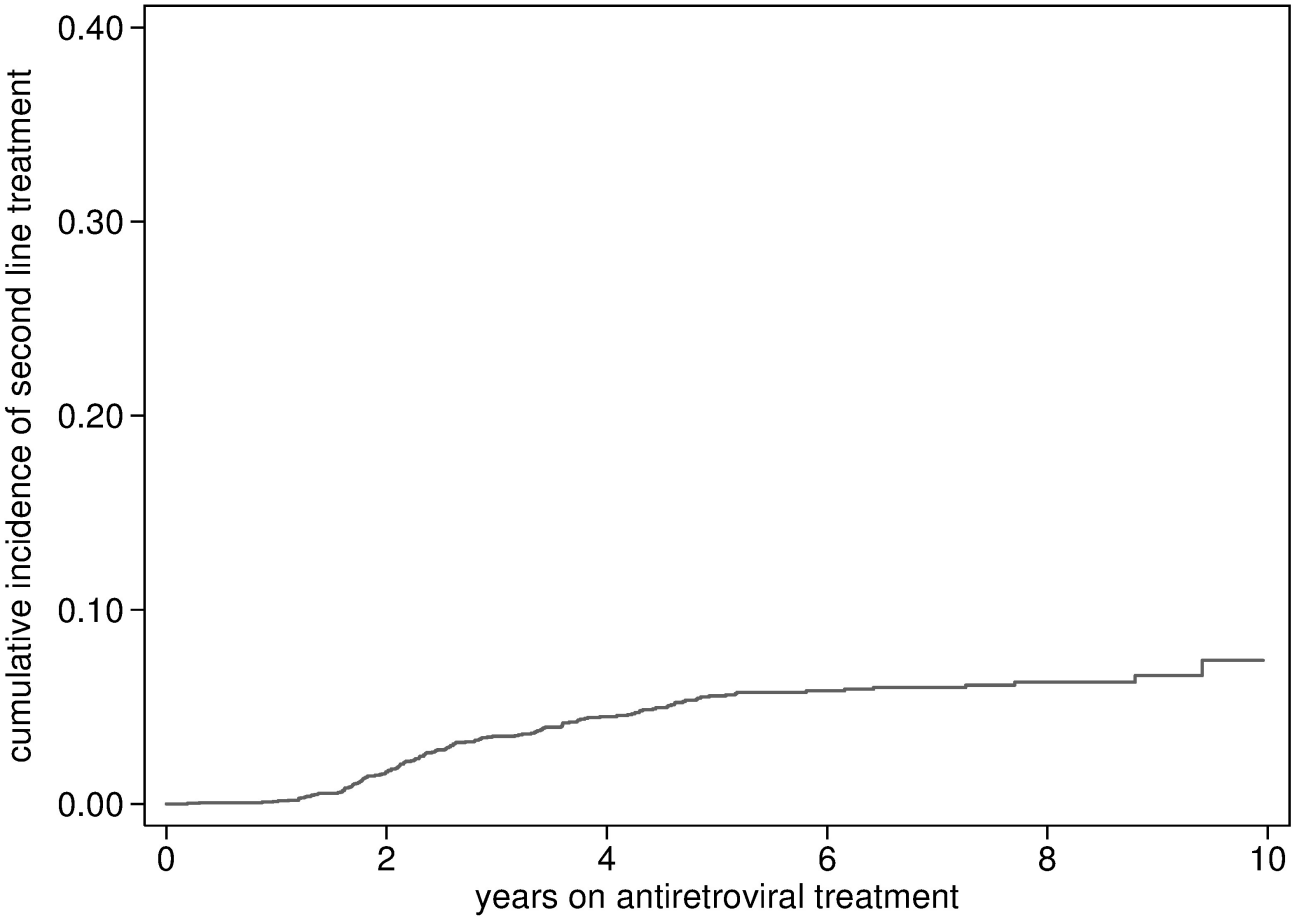
Cumulative incidence of first line treatment failure.

**Fig 8:**
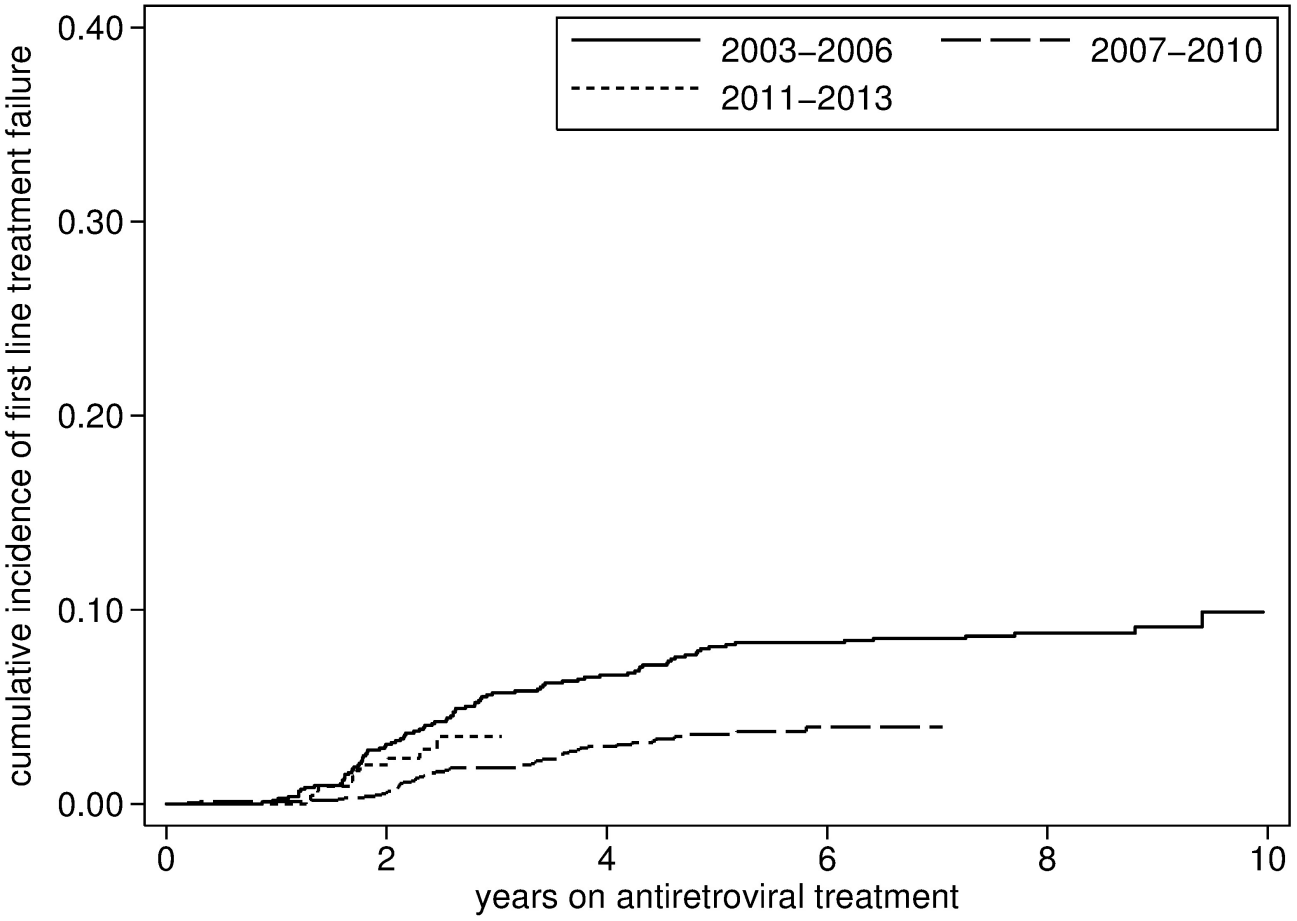
First-line antiretroviral treatment failure in Phnom Penh, Cambodia, stratified by period (2003–2014).

There were a total of 151 individuals with first line ART failure starting second line treatment, occurring a median of 4.8 years (IQR 2.4–7.1) after ART initiation. Of the 138 individuals whose file could be reviewed, a detectable viral load before was documented before the initiation of second line treatmentin 137; 135 had at least one viral load measurement above 1000 copies/ml. The probability of first line failure was estimated at 2.8% (95% CI2.3%-3.4%) at 3 years, 4.6% (95% CI: 3.9%-5.5%) at 5 years and 7.8% (95% CI 4.8%-12.5%) at ten years of ART (Fig 7). The probability was particularly high in the first few program years (Fig 8).

Factors independently associated with an increased risk of treatment failure included ART-experience, NVP-based ART and a baseline CD4 count below 200 cells/μL. Conversely, a lower risk was observed among individuals starting ART during the 2007–2010 period (Table 3).

## Discussion

This is one of the few studies reporting on longitudinal program outcomes over a ten year period in a setting with standardized patient management and prospective data collection, and whereby the consecutive revised WHO guidelines were readily implemented. Overall program outcomes were fair, and generally compare well to other reports from the region [21–26]. Even during scaling-up of the program, LTFU remained relatively low and stable. Mortality rates were higher during the first years of the program, when patients generally initiated ART with very advanced HIV disease. During that period, around two thirds initiated ART with a WHO clinical stage IV and/or a CD4 cell count < 50 cells/μL.

As a previous study from our hospital reported [27], while CD4 count levels at ART initiation gradually increased over time, there were no clear trend changes and infliction points concurrent with the different revisions of the WHO guidelines, recommending progressively earlier ART initiation. Since–particularly at the hospital level–individuals are still diagnosed relatively late with HIV, most with CD4 counts < 200 cells/μL increasing CD4 count thresholds will only impact on a small proportion of patients. Consequently, the impact of the revised guidelines will require effective efforts engendering early HIV testing. Similar observations have been reported from sub-Saharan Africa [3]. Early diagnosis should be combined with good access to and retention into HIV care. Of interest, we observed a decrease in CD4 count levels at ART initiation concurrent with a period of reduced access to and availability of HIV care at the national level. As our program continued to provide free HIV care without restricting enrolment, this decrease in CD4 count levels at ART initiation likely reflects difficulties for some patients in accessing HIV care elsewhere, ultimately attending our program with substantial delays.

The first-line failure rate was relatively low, and clearly below what has been reported in studies from Africa [9, 10, 28]. Since viral load testing was not done systematically throughout, a certain extent of underestimation is possibly in our study. Additionally, some patients with virological treatment failure might never have started second line treatment, for instance for reasons of death or LTFU. Nevertheless, our findings are in keeping with other reports/studies on Cambodian cohorts–including from our hospital–employing systematic viral load testing [18, 19, 21, 23]. Very low failure rates were also reported in a recent clinical trial from Thailand, also in the arm without systematic viral load monitoring [29]. Nevirapine use has been found to be associated with increased risk of first line ART failure in most observational and interventional studies [30]. ART-experience and a low baseline CD4 count have been found associated with first line failure in other studies as well [9, 21, 31].

The LTFU rate remained relatively stable over the ten year period, in contrast with some studies from South-Africa, whereby increased rates of lost to follow-up were seen concurrent with program scaling-up [9, 10, 28]. Although the comprehensive free HIV care provided in our hospital might have contributed to that, increasing rates of LTFU have been reported from other NGO-run programs providing free care. The implementation of patient tracing by telephonic contacting might also have contributed. Why individuals initiating stavudine were less likely to be LTFU remains to be defined. In our hospital, the stavudine-containing fixed dose regimen is dispensed for up to a three months period. Other less commonly used regimens, especially if containing efavirenz, are provided generally in limited quantity by the national program and hence are only dispensed for a maximum of two months in our hospital. The lower pill count or the more flexible dispensing of the stavudine-containing fixed dose regimen could possibly have led to better program retention. Although such perceptions were indeed occasionally expressed by patients in the program, corroborating this would require further study.

The clear–and possibly increasing–gap between males and females in CD4 count levels is concerning. Similar observations were made in a recent multi-country study in Africa [3] including one from our hospital [27]. While clear gains have been observed over the last few years for females, no such effect was apparent for males. Numerous factors could be involved, including health seeking behavior and increased health system contacts via PMTCT and maternal and child care services [32][33]. Nevertheless, the underlying factors remain to be urgently defined, to allow designing novel strategies to effectively reach, enroll and retain HIV infected males. Importantly, after adjusting for the baseline CD4 count, males remained at higher risk of death and attrition, as has also been observed in other studies [24, 34, 35]. This indicates that additional efforts beyond earlier ART initiation are required to improve outcomes amongst males.

One of the strengths of the study is the long study period, allowing to effectively implement the different revised WHO guidelines over the 10 years period, and the document changes over time in patient characteristics and outcomes. The hospital has also consistently been providing free ARV care, drawing patients from across the country. Main limitations include the single-center nature, limiting the generalizability. Nevertheless, as the only program consistently providing free care over the study period, with patients coming from across the country, our program data reflect to some extent changes occurring at the national level. Some degree of under ascertainment of deaths is likely. We also did not perform systematic viral load testing across the ten year period, but performed two cross-sectional evaluations on top of the routine monitoring. Detailed adherence information was lacking as well.

In conclusion, ART was started at progressively higher CD4 count levels across the ten year period. Nevertheless, in a hospital setting like ours, more than half still initiate ART with CD4 counts < 200 cells/μL. Earlier HIV diagnosis and enrolment into care will be required to achieve maximum impact of the move towards early initiation of ART. Despite advanced HIV disease, overall satisfactory patient and program outcomes were achieved during scaling-up.

## Supporting information

S1 Data (DTA)

S1 Fig Cumulative incidence of death, lost to follow-up and transfer-out after initiation of antiretroviral treatment. (TIF)
