# Supplementary figures and images for: Ten year experience with antiretroviral treatment in Cambodia: Trends in patient characteristics and treatment outcomes
Source: PLoS One. 2017 Nov 14;12(11):e0185348. doi: 10.1371/journal.pone.0185348 (PMC5685593; doi:10.1371/journal.pone.0185348)

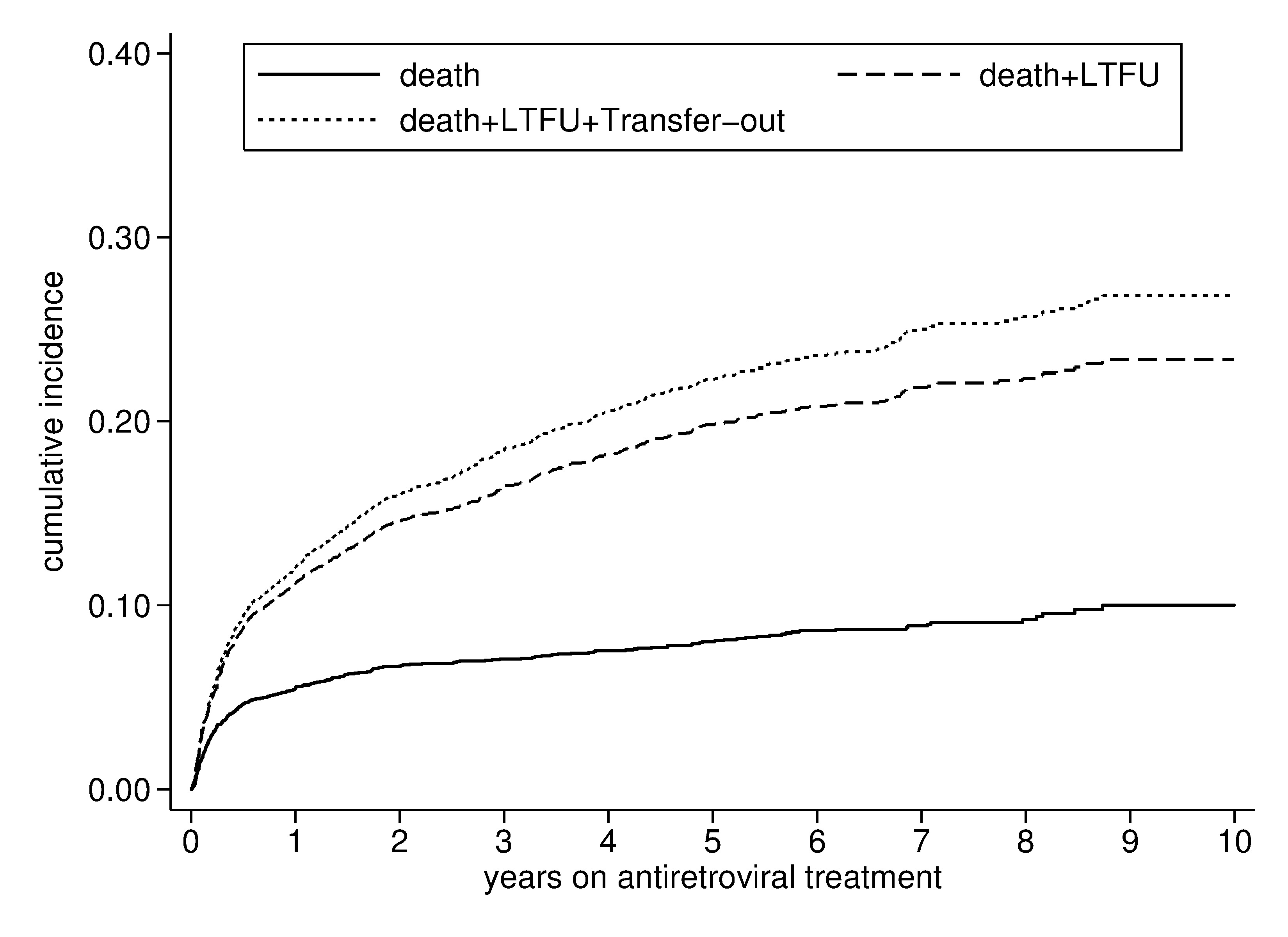

Supplement: S1 Fig — (TIF) [file pone.0185348.s002.tif]
